# Performance Evaluation of Monocular Markerless Pose Estimation Systems for Industrial Exoskeletons

**DOI:** 10.3390/s25092877

**Published:** 2025-05-02

**Authors:** Soocheol Yoon, Ya-Shian Li-Baboud, Ann Virts, Roger Bostelman, Mili Shah, Nishat Ahmed

**Affiliations:** 1 National Institute of Standards and Technology, Gaithersburg, MD 20899, USA; ya-shian.li-baboud@nist.gov (Y.-S.L.-B.); ann.virts@nist.gov (A.V.);; 2 Institute for Soft Matter Synthesis and Metrology, Georgetown University, Washington, DC 20057, USA; 3 Smart HLPR LLC, Troutman, NC 28166, USA; 4 Department of Mathematics, Albert Nerken School of Engineering, The Cooper Union for the Advancement of Science and Art, New York, NY 10003, USA; mili@cooper.edu; 5 Department of Electrical Engineering, Albert Nerken School of Engineering, The Cooper Union for the Advancement of Science and Art, New York, NY 10003, USA

**Keywords:** exoskeleton, pose estimation system, joint pose estimation, performance evaluation

## Abstract

Industrial exoskeletons (a.k.a. wearable robots) have been developed to reduce musculoskeletal fatigue and work injuries. Human joint kinematics and human–robot alignment are important measurements in understanding the effects of industrial exoskeletons. Recently, markerless pose estimation systems based on monocular color (red, green, blue—RGB) and depth cameras are being used to estimate human joint positions. This study analyzes the performance of monocular markerless pose estimation systems on human skeletal joint estimation while wearing exoskeletons. Two pose estimation systems producing RGB and depth images from ten viewpoints are evaluated for one subject in 14 industrial poses. The experiment was repeated for three different types of exoskeletons on the same subject. An optical tracking system (OTS) was used as a reference system. The image acceptance rate was 56% for the RGB, 22% for the depth, and 78% for the OTS pose estimation system. The key sources of pose estimation error were the occlusions from the exoskeletons, industrial poses, and viewpoints. The reference system showed decreased performance when the optical markers were occluded by the exoskeleton or when the markers’ position shifted with the exoskeleton. This study performs a systematic comparison of two types of monocular markerless pose estimation systems and an optical tracking system, as well as a proposed metric, based on a tracking quality ratio, to assess whether a skeletal joint estimation would be acceptable for human kinematics analysis in exoskeleton studies.

## 1. Introduction

Worker safety and health are core to sustainable manufacturing [1,2]. Awkward postures, where the body deviates significantly from the neutral position while performing work, are known to cause work-related musculoskeletal disorders (WMSDs) [3]. In 2018, the WMSD incident rate was 30.6% in U.S. manufacturing industries [4]. To reduce worker fatigue and the rate of WMSDs, industrial exoskeletons are evolving and have demonstrated benefits when applied to workers in automotive [5], aircraft [6], shipbuilding [7], and construction industries [8]. To effectively utilize industrial exoskeletons, it is important to understand how workers are supported in their work pose and how musculoskeletal fatigue can be reduced using exoskeletons.

A standard exoskeleton evaluation framework could be beneficial to understanding the effects of using exoskeletons. Such standard evaluation frameworks can comprise application area, wearer activities, task type, tasks involving joints, and other defined measurement parameters in the utilization of the exoskeleton, such as functional, ergonomic, task performance, and usability metrics [9]. The ASTM Committee F48 on Exoskeletons and Exosuits has published standard practices for exoskeletons. The ASTM F3443-20 Standard Practice for Load Handling When Using an Exoskeleton provides test methods to evaluate an exoskeleton for a load handling task [10] and was applied to tests for measuring physical exertion with respect to gender, anthropometry, and fit [11]. Similar test methods for evaluating exoskeletons for peg-in-hole assembly [12] and applied force have been created [9]. The results from these test methods can be used to determine how exoskeletons can best be used to support the user [9].

The ASTM F3518-21 Standard Guide for Quantitative Measures for Establishing Exoskeleton Functional Ergonomics Parameters and Test Metrics [13] describes measurements for assessing the ergonomics of exoskeletons. The quantitative measures for evaluating the ergonomics of exoskeletons include, but are not limited to, electromyography, motion capture, task completion time, pressure mapping, 3D volumetric changes, metabolic rate, strength, and heart rate [13]. This study proposes a methodology to evaluate monocular markerless pose estimation algorithms to enable the assessment of ergonomic performance, task performance, cognitive effects, or physiological changes with and without the use of an exoskeleton by obtaining pose estimation data while performing 14 pre-defined industrial poses.

Joint kinematics [12,14] and joint angles are common ergonomics metrics. Joint angle changes include knee flexion, back rotation, back flexion, trunk flexion, arm flexion, and shoulder flexion [15]. ASTM F3474-20 Standard Practice for Establishing Exoskeleton Functional Ergonomics Parameters and Test Metrics also defines range of motion, degrees of movement, kinematics, and task completion time as functional ergonomic metrics that can be derived from joint pose data [16]. Table 1 summarizes research to measure joint activity changes while wearing an exoskeleton and performing a task. Optical tracking systems (OTSs) (also called motion capture systems) and inertial measurement units (IMUs) are widely used to measure joint angles. IMU repeatability (i.e., the root mean square error for various joints and planes of motion) can range from 1 degree to 6 degrees [17,18]. Another study observed no significant difference in joint kinematic data for knee and pelvis flexion, while hip flexion was reduced when using IMUs compared to an OTS [19].

Monocular markerless pose estimation systems based on color (RGB) and depth cameras augmented with deep learning image processing technologies are being evaluated for use in exoskeleton studies [25]. Markerless pose estimation systems provide estimated joint poses based on relative two-dimensional (2D) or three-dimensional (3D) position and quaternion rotations, from the captured RGB or depth images [26,27]. RGB cameras have additional advantages due to their low cost compared to OTS and IMU technologies, ubiquity, ease of use and maintenance, data interpretability, and deployment flexibility in both laboratory and industrial environments [25]. A markerless system alleviates subjects from having to wear sensors, a motion capture suit, or attached markers, all of which can impact a user’s movement and cognitive state [28,29]. Test subjects without additional sensors have the potential to improve exoskeleton kinematic measurement fidelity.

The performance of markerless pose estimation systems depends on various factors. Table 2 summarizes the studies on the performance of markerless pose estimation systems compared to a reference system, such as an OTS or an IMU. Each study defined identical conditions and constraints during the experiments, including joints of interest, subject pose, and sensor placement. It has been shown that viewpoint (the distance and angle from which the camera views and records the subject), occlusion, image resolution, and subject pose are some examples of factors affecting markerless pose estimation performance.

**Figure 1:**
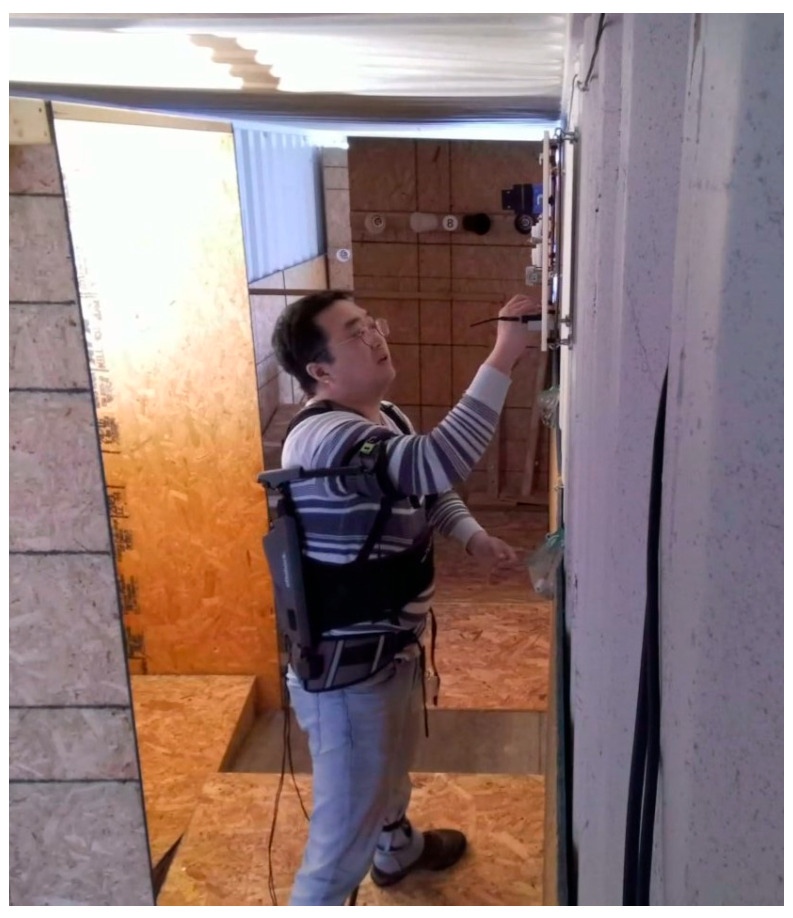
Image of a subject performing simulated wiring tasks while wearing a shoulder exoskeleton. The exoskeleton occluded joints on the upper arm, the back, and the right hip.

**Figure 2:**
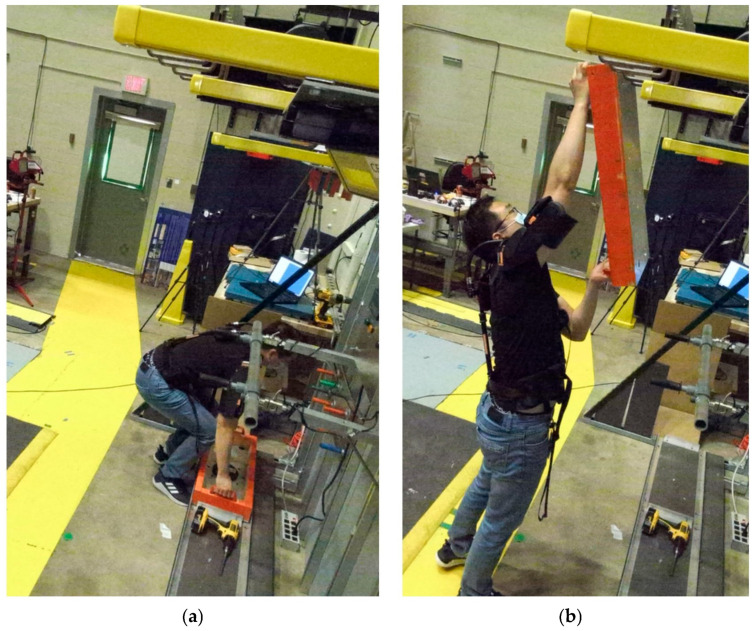
A subject performing a load positioning task simulating material handling in the manufacturing industry while wearing a shoulder exoskeleton. The task begins with (**a**) lifting the load from the ground, providing a view of back and knee flexion from the sagittal plane. The task ends with (**b**) hanging the load overhead, requiring arm extension and flexion.

Viewpoint, wearable robot, and subject pose are the factors considered in this study. The selected factors can contribute to pose estimation errors for industrial exoskeleton performance evaluation. While performing industrial tasks, workers interact with parts, products, walls, the floor, or the test apparatus [11]. As work environments need to be optimized for task efficiency and worker safety, image sensors may be limited to the side or back, whereas pose estimation performance is often optimized for front or side-front sensor placement [41]. Because of viewpoint limitations, occlusions can occur due to the subject themselves, and wearing an exoskeleton can result in further occlusions. When a task such as grinding requires a tool, the tool can also cause occlusions. Figure 1 demonstrates a viewpoint with occlusions. Unlike the studies that defined target tasks with a few joint movements, many industrial tasks require full-body motion, such as lifting, holding, carrying, dragging, kneeling, bending, reaching overhead, and crawling [16]. Figure 2 shows examples of industrial tasks with simultaneous motions of the legs, lower back, and arms. Therefore, there is a need to evaluate the pose estimation performance for a full body.

**Figure 3:**
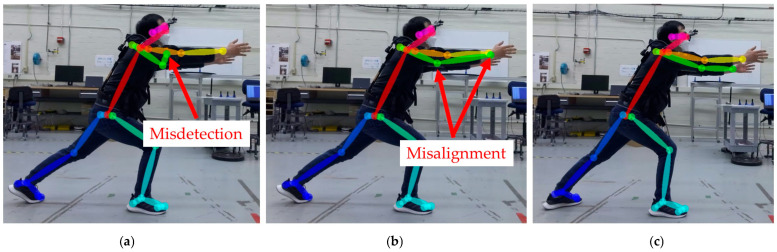
Pose estimation result showing (**a**) a misdetection, where the left arm posture is incorrect; (**b**) a misalignment, where the detected joints on the left arm are not aligned with the actual joints; and (**c**) a correct detection, where the detected joints appear to coincide with the subject’s joints.

Several studies evaluated the joint poses indirectly—for instance, comparing a joint angle or a step distance to a reference motion capture system to determine the relative difference in joint detection and joint angles. Prior to evaluating markerless pose estimation methods, a methodology to curate the images with missed joint detection is proposed for evaluating both joint position and relative angle changes. Joint detection and position errors can be categorized into misdetections (Figure 3a) and misalignment (Figure 3b). Misdetection refers to when the estimated joint pose is out of the subject’s body, or the nearest joint from the estimated joint pose is not the target joint. Misalignment refers to when the detected joint is on the subject’s body but the detected joint deviates from the actual joint. A correct detection refers to a case where the skeletal joints appear to coincide with the subject’s joints (Figure 3c). By excluding results with misdetections or misalignments, both of which can be determined by visual inspection or with automation using a computational algorithm, joint position errors can be statistically measured via comparison with a reference system.

Although an OTS is commonly used as a reference system in body-tracking studies, extra care is needed when applied to industrial exoskeleton evaluations. ASTM F3518-21 defines a motion capture system (or OTS) as a tool for quantitative ergonomics measures, where it is imperative to place retroreflective markers accurately on anatomical landmarks [13]. However, there may be cases where the positions of the retroreflective markers and the exoskeleton overlap. If a marker is placed on a motion capture suit, the exoskeleton may obstruct the cameras’ view of the marker. If the marker is placed on the exoskeleton, the pose estimation performance degrades as the kinematics of the human body and the exoskeleton differ [14]. In either case, misdetection or misalignment of the actual subject’s joints can also occur in the OTS measurements.

To improve pose estimation performance and evaluate the effects of wearing an exoskeleton on the users’ posture, clarifying when and how misdetections and misalignments occur is important in order to properly curate data for analysis. The research should be performed first on monocular pose estimation systems, as multi-view pose estimation systems have mainly been developed for building 3D poses [42], relying on each monocular pose estimation performance [43]. A preliminary study of monocular markerless pose estimation performance was conducted to understand the interactions and impacts of exoskeletons, sensors, viewpoints, joints of interest, and task poses on human pose estimation [25]. The results showed that pose estimation performance using depth cameras had a 38% acceptance rate, compared to a 91% acceptance rate for the OTS, where the pose estimation result was accepted by the criteria to be described in Section 2.4. It was also shown that the joint pose error depends on the joints of interests and task poses for both systems [25].

The purpose of the study is to understand how markerless pose estimation system performance changes based on the wearable robot, viewpoint, and industrial task pose. Sources of joint detection errors, potential applications using current monocular markerless pose estimation systems, and augmentation of pose estimation algorithms for simultaneous human and exoskeleton joint detection will also be discussed. The primary contribution of this study is to provide a methodology to curate images produced by monocular markerless pose estimation systems in the context of using industrial wearable robots. The curated images can then be applied for further analysis to estimate joint pose errors between a markerless pose estimation system and a reference system. This study is a continuation and an extension of the conference paper published in 2022 [25].

## 2. Experimental Method

An experiment with a factorial design of 14 industrial task poses, 10 viewpoints, 2 image types, and 3 types of exoskeletons was conducted. The subject executed different industrial task poses with the cameras positioned at various viewpoints with respect to the subject. The estimated joints, computed offline from the RGB and the depth images, were evaluated to determine whether the resulting poses had misdetections or misalignments. The OTS pose estimation was evaluated and compared as the reference system. The experiment consisted of three steps. First, images were collected from RGB and depth sensors. Second, joint poses were estimated via pose estimation software. Third, the estimated joint poses were evaluated in terms of whether their results showed misdetections or misalignments. In addition, tests without an exoskeleton were used as the control to specifically gain insight into the impact of wearable robots.

The experiment was conducted by a single subject to reduce confounding parameters. The subject fits the body specifications of the exoskeletons. The subject wore the same clothes and shoes for each test to minimize measurement variability.

### 2.1. Poses

**Figure 4:**
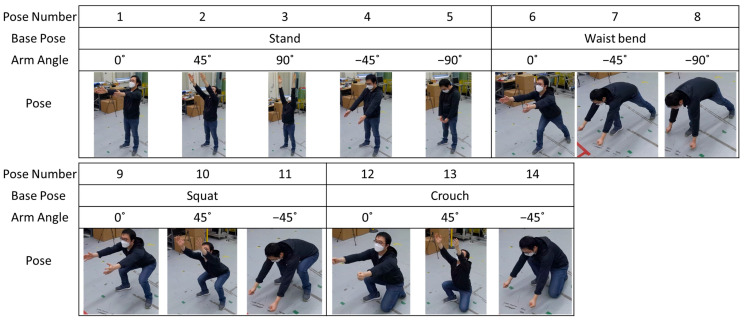
Target industrial task poses.

Fourteen industrial task poses were defined based on observed task poses from exoskeleton performance tests for industrial tasks, including load handling, peg-in-hole, load alignment, and applied force [11,12,44]. Each pose is a combination of base poses and arm angles. Base poses represented the following four positions: stand, waist bend, squat, and crouch. Arm angles included 0°, 45°, 90°, −45°, and −90°, where the arms stretched forward, forward-up, up, forward-down, and down, respectively. Figure 4 shows the subject performing each task pose.

### 2.2. Types of Exoskeletons

**Figure 5:**
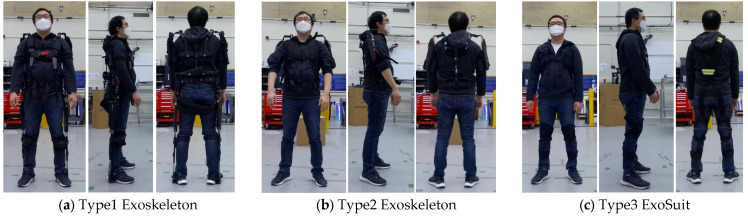
The subject wore (**a**) a full-body exoskeleton, (**b**) a shoulder exoskeleton, and (**c**) an exosuit.

Three types of exoskeletons were used in this experiment. Type 1 is a full-body exoskeleton. It consists of a rigid metal frame with straps on the legs, the hips, the back, and the shoulders. Type 2 is a shoulder exoskeleton. It consists of a rigid metal frame and soft straps on the back and shoulders. Type 3 is an exosuit composed of soft elastic straps and padding, supporting the back for standing, squatting, and lifting, with elastic bands on the leg and back, and safety reflective tape on the back and shoulders. Figure 5 shows the three exoskeleton types.

### 2.3. Sensor and Image Capture

A Microsoft Azure Kinect Camera [27] was used as an image capture device. (Disclaimer: Certain commercial equipment, instruments, or materials are identified in this paper to foster understanding. Such identification does not imply recommendation or endorsement by the National Institute of Standards and Technology, nor does it imply that the materials or equipment identified are necessarily the best available for the purpose). The camera captures color and depth images together into a single file stream. The color image resolution is 1920 × 1080 pixels and the depth image is an unbinned narrow field of view (NFOV) with a resolution of 640 × 576 pixels. The images were captured at 30 frames per second. OpenPose v1.5.1 [26] was used for the RGB pose estimation and the Azure Kinect Body Tracking Software Development Kit (SDK) v1.0.1 [27] was used for the depth pose estimation.

**Figure 6:**
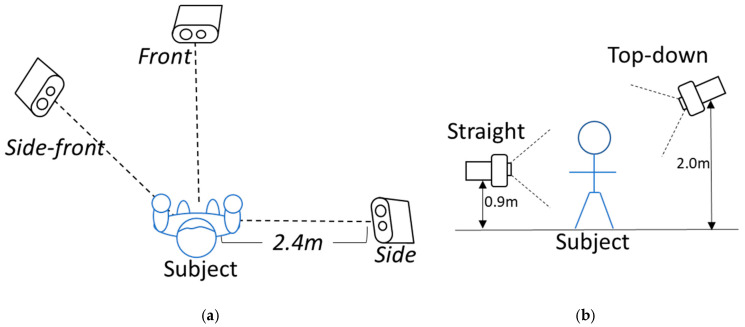
The (**a**) camera placement as viewed from above the subject, and (**b**) height adjustment as viewed in front of the subject.

Three synchronized cameras were placed facing the subject. Camera positions, nominally 2.4 m away from the subject, were front, side-front, and side. Camera height and angle relative to the subject were straight at 0.9 m and 0.0°, and top down at 2.0 m and 18.0°. Figure 6 shows the camera placements (a) and height adjustments for different viewpoints (b).

For each target task pose and exoskeleton, the images were captured four times for each position (for a total of ten different viewpoints) using (1) a straight view with the subject facing the front camera, (2) a straight view with the subject facing the opposite side of the front camera, (3) a top-down view with the subject facing the front camera, and (4) a top-down view with the subject facing the opposite side of the front camera.

**Figure 7:**
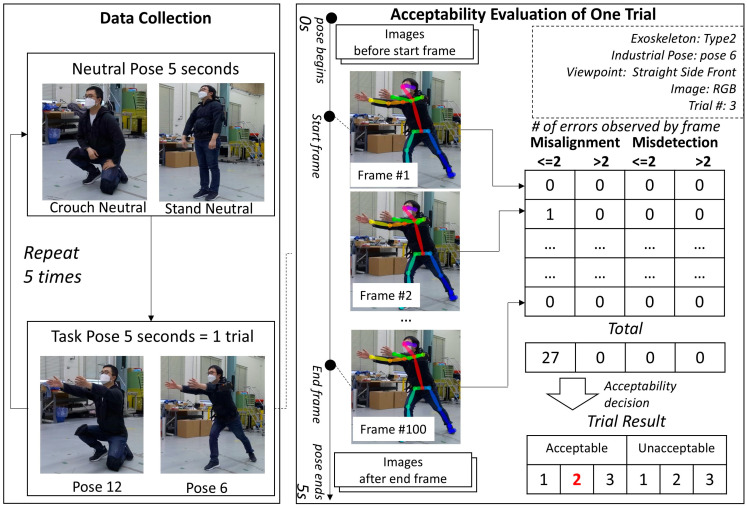
Procedure for markerless pose estimation data collection and evaluation.

For each exoskeleton, pose, and viewpoint, images were collected using the following procedure. The subject began each trial in either a crouching or a standing neutral pose, as shown in Figure 7. For industrial crouching poses, the subject began in a crouching neutral position. For standing, bending, and squatting industrial poses, the subject began in a standing neutral position. Second, the subject held one of the 14 industrial task poses for 5 s. Third, the subject returned to a neutral standing pose. The subject repeated the task pose and the neutral pose five times. Out of 150 collected frames, 100 frames were selected for the evaluation of each trial. Beginning and end frames were not used, as the subject sometimes moved at the start and end, and especially when wearing an exoskeleton. Figure 7 describes the data collection and evaluation procedure.

### 2.4. Pose Estimation Evaluation

As discussed in Section 2.1, the poses are intended to simulate human poses while performing industrial tasks such as load handling, peg-in-hole assembly, load alignment, and applied force. Accordingly, this study defines joints of interest as the center of the shoulders, shoulders, elbows, wrists, center of hips, hips, knees, and ankles. Although the Type 2 and Type 3 exoskeletons support different joints, the joints of interest remain the same because the objective of the analysis is to measure and understand the extent to which an exoskeleton induces changes in body posture.

**Figure 8:**
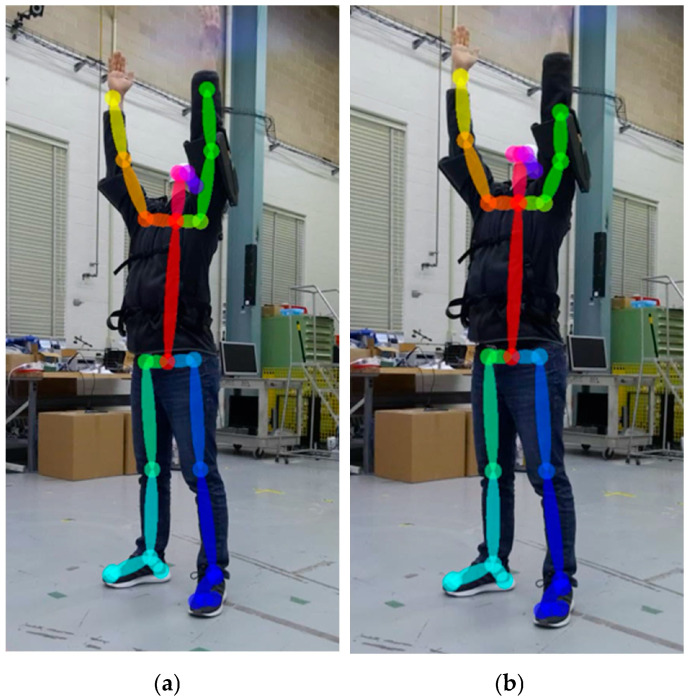

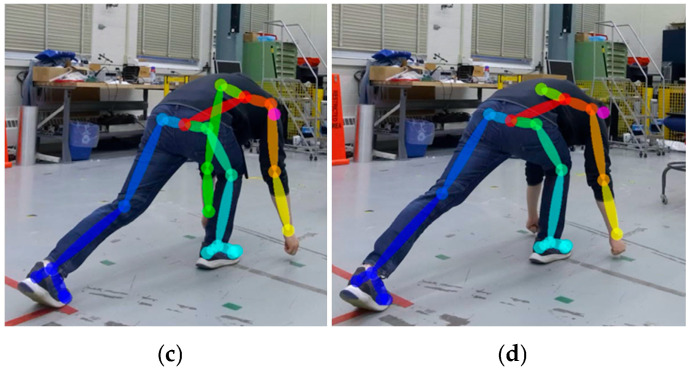
Examples of different results for the same test condition. The estimated left wrist joint can be nearest to (**a**) the wrist or (**b**) the elbow. The left arm can be (**c**) detected or (**d**) not detected.

The purpose of the evaluation is to determine whether the skeletal joint estimation in the sets of images from each trial includes misdetections or misalignments and to decide whether the image set from each trial can be accepted for further data analysis. Since no software tools to determine misdetection and misalignment of pose estimations are known, the result was evaluated visually by the researcher. As described in Section 2.3, only 100 images were used for a single iteration. The evaluation results can differ over 100 images. Table 3 shows the evaluation examples of three different conditions, and Figure 8 shows how different results can occur for the same condition.

Based on the experience in prior exoskeleton performance studies [9,12], the pose estimation results with misdetection or misalignment in trials can be used in some cases. This study set 80% as the threshold to accept the result, because it was observed (in a previous study [12]) that industrial task performance can be measured when at least 80% of the subject’s body is successfully tracked. Table 4 describes the cases where the pose estimation results are acceptable. Acceptable Case 1 (A1) is when the result is clearly acceptable. Acceptable Case 2 (A2) is when one or two linked joints show visible misalignment in the iteration. Acceptable Case 3 (A3) is when some joints have misdetection in the iteration. Unacceptable cases, U1, U2, and U3, are also defined in a similar way as shown in Table 4. Minor case handling is discussed in the next section.

Applications that can utilize A2 and A3 frames despite misalignment and misdetections will be described in the Discussion section.

### 2.5. OTS as the Reference System

An OptiTrack OTS was used as a reference system to compare the pose estimation performance [45]. The same image capture procedure as discussed in Section 2.4 was applied for the 14 poses and the 3 exoskeletons, as well as for the control test.

**Figure 9:**
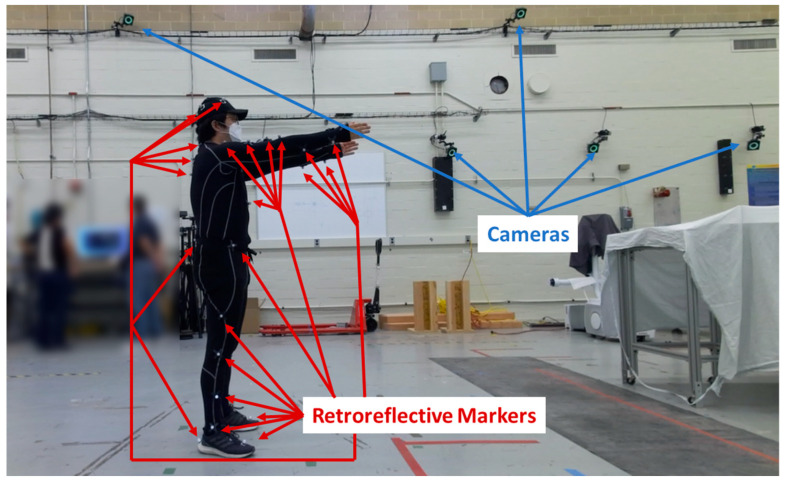
Retroreflective markers and optical sensor setup. The testbed laboratory includes 20 optical tracking cameras.

**Figure 10:**
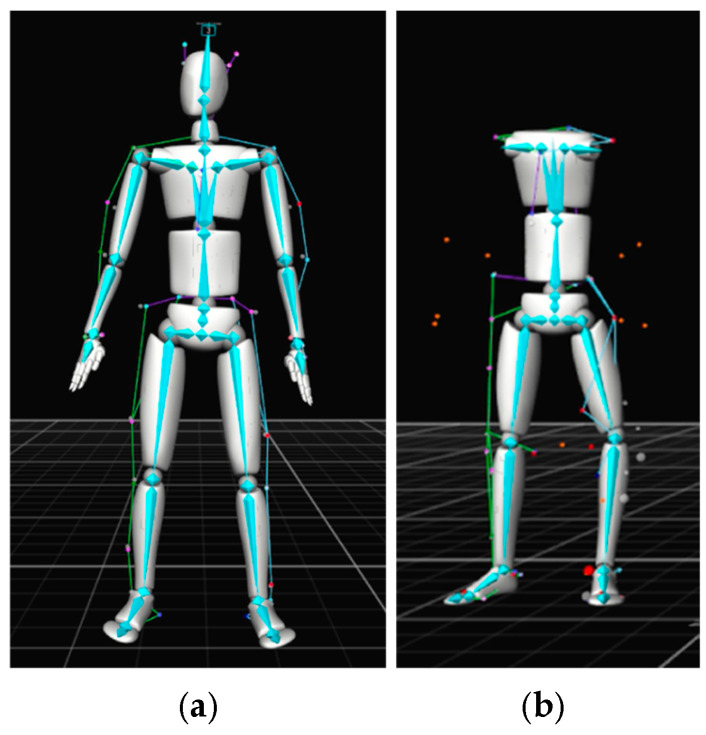
The (**a**) conventional (39 markers) and the (**b**) Rizzoli (43 markers) marker set models from the optical tracking software.

A total of 20 optical tracking cameras were mounted to walls, with some at a height of 2.7 m and others at a height of 4.3 m off the ground, within a 9 m wide × 22 m long × 7 m high test area. Data were acquired at a rate of 120 frames per second using Motive Software Version 1.10 [46]. The subject performed the test in the center of the test area. Figure 9 shows some of the cameras and the subject wearing the motion capture suit with passive retroreflective markers. Based on pre-programmed biomechanical skeleton marker sets, the retroreflective markers were attached to the suit, hat, shoes, and exoskeleton (when the exoskeleton frame or straps occluded the anatomical landmarks). The conventional skeleton marker set [47] was used for the control and Type 1 exoskeleton, while the Rizzoli skeleton marker set [48] was used for the Type 2 and Type 3 exoskeletons based on the inability to track the subject using the conventional model. The Rizzoli marker set could be constructed for skeletal tracking despite the adjustments to the skeletal marker sets to accommodate the occlusions caused by the exoskeletons. Additional markers were attached to the arms so they could be manually estimated. RGB videos were recorded to evaluate the OTS pose estimation results. Figure 9 shows the setup for optical motion capture, and Figure 10 shows the skeletal tracking result with the conventional and the Rizzoli marker sets.

## 3. Results

### 3.1. Pose Estimation Evaluation Results

A total of 5600 trials, as described in Figure 7, were conducted. Of these, 5577 evaluation results were retained, and 23 trials were excluded because of missing data during data collection and/or processing. There were cases where just one or two frames had misdetection or misalignment of 100 frames. Those frames were regarded as outliers and excluded when evaluating the iteration.

There were 2520 acceptable results (as described in Table 4) out of the 5577 evaluation results that were retained. Table 5 shows the result of the front, straight view as an evaluation example. Table 6 summarizes the result by the viewpoints. The front and side-front showed the largest acceptable results with a straight view, followed by the top-down views. The side and side-back with a straight view showed less than 30% acceptability. Many frames were categorized into U3, where one or two connected joints were incorrectly detected. Side, side-back, and back with a top view had improved acceptability rates of 33.6, 24.6, and 30.0%, respectively, compared to straight views of 24.0, 18.4, and 21.3%, respectively. The back view showed the largest U1 incorrect detection results, where more than two joints were incorrect in both the straight and the top view. Table 7 summarizes the result by exoskeleton and image type. From the monocular RGB camera-based pose estimation, all exoskeletons, as well as the control, showed A1, where the acceptability rate was between 39.1% and 44.1%. U3 had the second highest incorrect detection frequency for RGB images among all test cases, above 20%. Type 1 and Type 3 exoskeletons showed decreases in A1 correct detection results compared to the control. From the depth body estimation, U1 was shown mostly for all exoskeletons and the control to be between 37.4% and 62.3%. There were less correct detection results of A2 and A3 or incorrect detection results of U2 compared to the RGB results. Type 1 exoskeleton showed the lowest number of acceptable cases.

### 3.2. Acceptable Results by Category

This section describes the number of acceptable pose estimation results (A1, A2, and A3) arranged by the exoskeletons, the task pose, and the viewpoints. Table 8 describes the number of acceptable results by the exoskeleton type versus the pose. Pose 2 showed the best performance, with an acceptability rate of 71.5%. Poses 8 and 14 were shown to be the most difficult to estimate, with acceptability of 13.0 and 14.4%, respectively. There were cases where monocular pose estimation from one type of exoskeleton showed better or worse performance for a specific pose. For example, a subject with a Type 3 exoskeleton performing industrial task pose 6 performed better than the other cases. Pose estimation based on the depth camera was observed to be more susceptible to the subject pose, especially for poses requiring the subject to stretch their arms to the ground (poses 8, 11, and 14).

Table 9 describes the number of acceptable results by exoskeleton type and by viewpoint. From the monocular RGB camera-based pose estimation, the Type 1 exoskeleton showed more than 10% reduced acceptability from the back straight viewpoint, compared to the control (i.e., no exoskeleton) and other types of exoskeletons. The Type 1 exoskeleton showed increased image acceptability in the front and side-back straight view and the front top-down view compared to the control. The Type 2 exoskeleton showed at least a 6.9% reduced acceptability from the side-front top-down viewpoint, compared to the control and other types of exoskeletons. The Type3 exoskeleton showed 100% acceptability from the side-front straight viewpoint, but showed more than 10% reduced acceptability from the front straight viewpoint. From the depth camera-based body estimation, all the exoskeleton results showed lower than 20% acceptability from the side, side-back, and back viewpoints, including three cases of zero correct estimations. The Type 2 exoskeleton showed increased acceptability from the front straight viewpoint, and the Type 3 exoskeleton showed the closest acceptability rate to the control from the side-front viewpoints.

There were cases where the pose estimation performance significantly decreased compared to the control. The cases with a more than 10% decreased acceptability rate are highlighted in red in Table 9. The ide-back top-down viewpoint is shown to be the most challenging for all types of exoskeletons. From the monocular RGB camera-based pose estimation, the Type 3 exoskeleton showed the most decreased performance from the side-back top-down view, with an acceptability rate of 34.3% compared to the control’s 55.7%. From the depth camera-based pose estimation, the Type 2 exoskeleton showed the most decreased performance from the side-front straight focal point, with an acceptability rate of 37.1% compared to the control, with 64.3%. There were cases where the pose estimation performance increased compared to the control. The monocular RGB camera-based estimation of the Type 1 exoskeleton from the side-back straight and front top-down focal points showed more than a 10% increase in acceptable frames, as demonstrated in bold in Table 9.

Table 10 describes the number of acceptable results for the viewpoint versus the industrial pose. The pose estimation from the straight viewpoint had the lowest number of acceptable frames when the camera was placed at the side, side-back, and back relative to the subject, and while the subject performed industrial tasks involving bending at the waist (poses 6, 7, and 8), arms forward or downward with squat (poses 9 and 11), or crouch (poses 12 and 14). The combinational effect between pose and viewpoint is highlighted in red in Table 10. The frames from the side top-down camera did not show the combinational effect except for crouching poses. There were other combinations where the monocular pose estimation performance was significantly lower, such as when the frames included cameras placed at the front straight, with the subject performing pose 8, or when the cameras were placed at the side-back or back straight, with the subject performing pose 1.

Table 11 describes the number of acceptable pose estimation results by exoskeleton type and industrial pose type using a reference OTS. There were incorrect estimations of pose 11 from the control test. Frames with the subject wearing a Type 1 exoskeleton showed the lowest number of acceptable pose estimations. Frames with the subject crouching (pose 14) while wearing a Type 2 exoskeleton showed a decrease in acceptable pose estimations, and a single incorrect estimation in pose 8 and pose 11. The Type 3 exoskeleton showed the most acceptable skeletal joint pose estimations for all trials.

## 4. Discussion

### 4.1. Factors Contributing to Pose Estimation Errors

Differences in pose estimation performance were observed between different industrial task poses, viewpoints, and exoskeleton types. This section discusses the factors affecting pose estimation observed during the experiment, mainly due to occlusions by the subject while performing an industrial task pose, and to the presence of the exoskeleton.

#### 4.1.1. Occlusions

**Figure 11:**
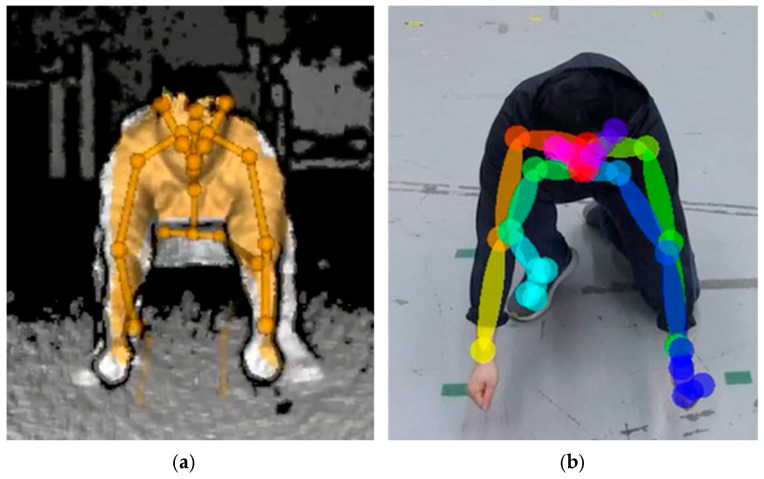
Occlusions caused by the task pose: The shoulders and arms can occlude the joints on the hips and the legs when performing (**a**) squatting and (**b**) crouching poses.

Occlusions remain a major contributor to human pose estimation errors [49]. In this study, occlusions were caused primarily by the task poses and the viewpoints. Table 8 shows how the occlusions by the pose affected the body estimation performance. When the arms are outstretched, towards the ground, the arm or leg can become indiscernible or invisible to the sensor. Poses 7, 8, and 11 showed the lowest number of acceptable pose estimations compared to the other poses, because the arms were placed in a downward position, occluding the lower-body joints. When the joints of interest were in closer proximity, some joints would cover the other parts of the body. Pose 5 puts the wrists near the hips, where the wrists can be seen only by the front or side-front viewpoints. Crouching poses align the ankle and the hip together, and the ankle was rarely estimated correctly. Pose 14 scored the worst due to occlusions from the arm during the crouch position. Figure 11 demonstrates the joint estimation errors caused by the task pose occlusions.

**Figure 12:**
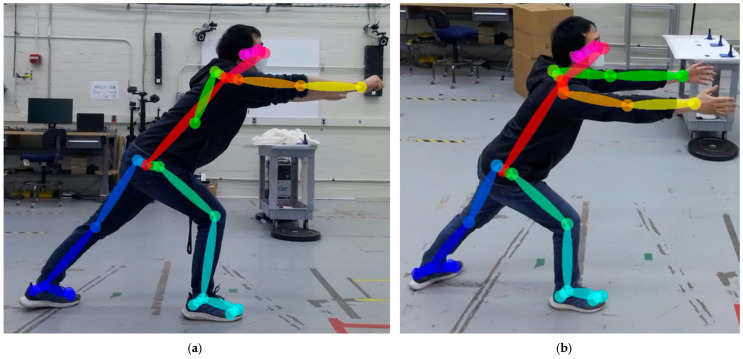
The left arm visibility from the side when the (**a**) angle is straight and (**b**) the angle is vertically tilted from the top down for the subject performing industrial pose 6.

Table 9 shows how the occlusions due to viewpoint affected the body estimation performance. When the images were taken from the side, the arm, leg, or both on the other side would be invisible to the sensor. When the images were taken from the back, the arms would be invisible unless the task involved overhead work, where the arms would be in an upward position. From the back, the knees could become less discernible unless the subject was in a standing position. The camera’s vertical angle could also change the visibility of the joints. Figure 12 shows how the pose estimation changes depending on the camera’s vertical angles.

**Figure 13:**
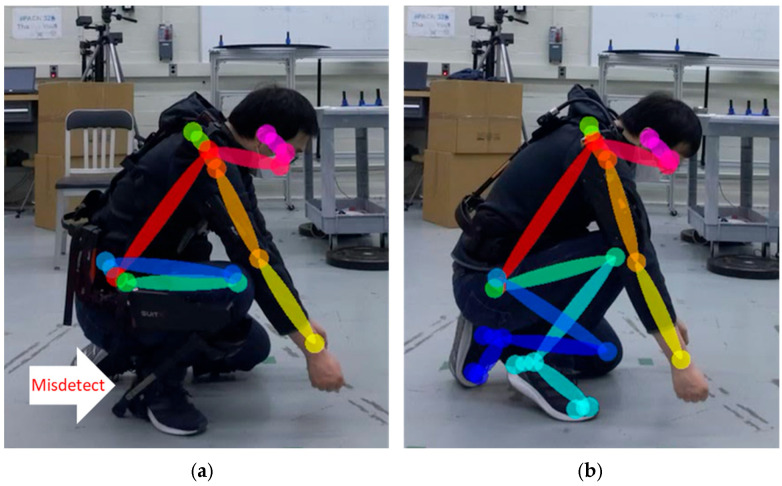
The right ankle is incorrectly estimated due to the (**a**) Type 1 exoskeleton covering the right ankle joint compared to (**b**) the Type 2 exoskeleton, where the joint is visible.

Wearing exoskeletons can further contribute to occlusions. The exoskeleton increases the anthropometric footprint of the human frame, thus, on occasion, covering one or more joints. This makes detecting the skeletal joints challenging for typical pose estimation algorithms. Figure 13 shows incorrect joint estimations due to occlusion by the exoskeleton frame.

The occlusions discussed here should be considered when applying markerless pose estimation methods in industrial exoskeleton studies. The joints of interest may be covered by other body parts, especially for industrial poses when the joints are in proximity. In addition, sensors may not be placed in the optimal position to avoid high human–workpiece interaction areas. Lastly, there could be additional occlusions by the tools, products, or worktables.

#### 4.1.2. Wearing Exoskeletons

Wearing an exoskeleton changes the subject’s appearance. There were remarkable changes in the pose estimation results in certain cases, as shown in Table 7. For the monocular RGB camera-based pose estimation, the number of frames adhering to unacceptable cases increased, while the number of frames adhering to A1 and A3 acceptance decreased, meaning the study observed an increase in misdetections or misalignments when wearing an exoskeleton. For the depth camera-based joint estimation, the number of frames adhering to A1 increased, while the others decreased or remained the same, meaning the study observed an increase mostly in misdetections when wearing an exoskeleton.

**Figure 14:**
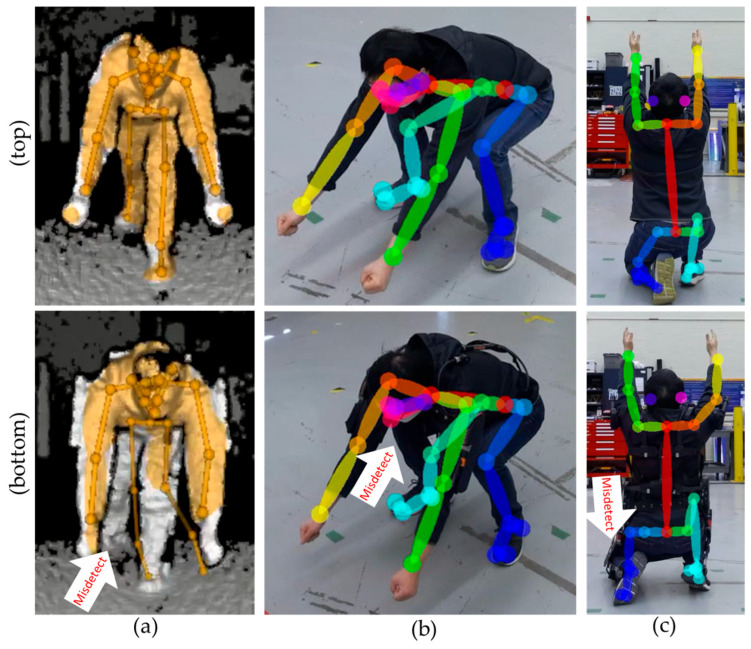
Incorrect joint pose estimation when wearing exoskeletons (bottom) compared to the control (top). The target joints are visible but misdetected: (**a**) ankles, (**b**) right knee, and (**c**) left knee.

**Figure 15:**
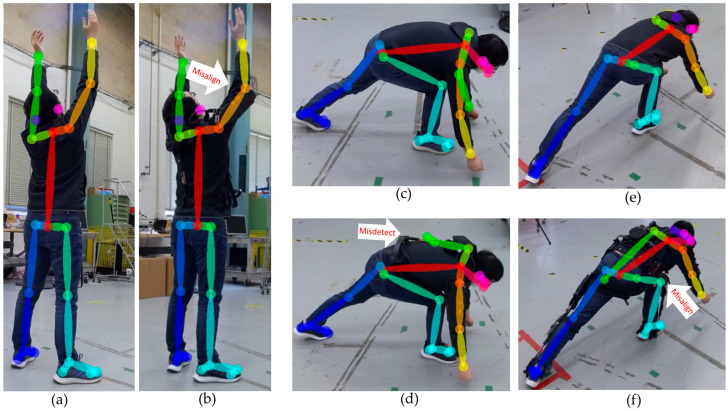
The exoskeleton part is detected as (**b**) the right elbow, (**d**) left arm, and (**f**) right knee compared to the control (**a**,**c**,**e**).

**Figure 16:**
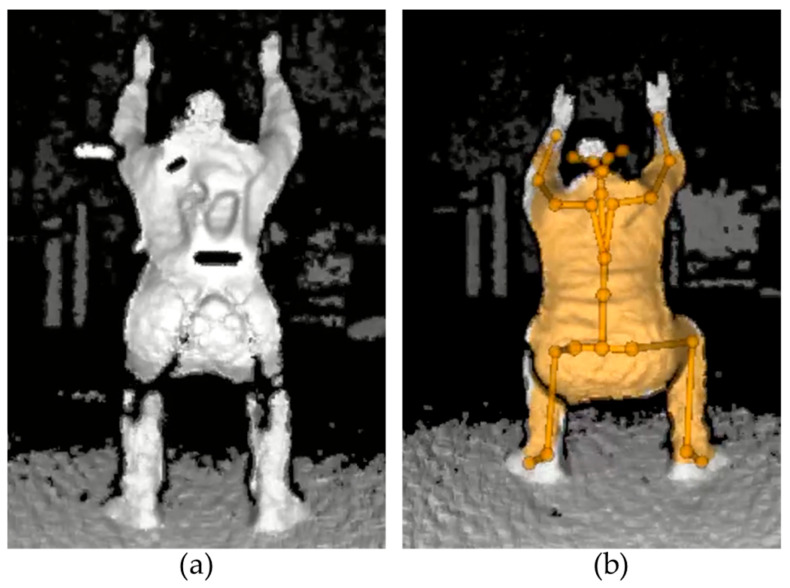
Skeletal joints were not detected while wearing the exoskeleton (**a**) compared to the control (**b**).

The effects exoskeletons have on human joint estimation often appeared as missing or incorrect joint detections. Figure 14 shows cases where incorrect joint estimation occurred in which the joint was visible. From the monocular RGB camera-based pose estimation system, the exoskeleton frames were sometimes confounded with body parts. The most common cases were when the upper arm frame was detected as a shoulder or elbow joint, the back frame was detected as an arm joint, and the leg frame was detected as a knee joint, as shown in Figure 15. From the depth camera-based pose estimation system, the Type 3 exoskeleton caused the sensor to work improperly due to reflective tape, resulting in failure to detect any of the human joints, as shown in Figure 16.

### 4.2. Reference System Analysis

**Figure 17:**
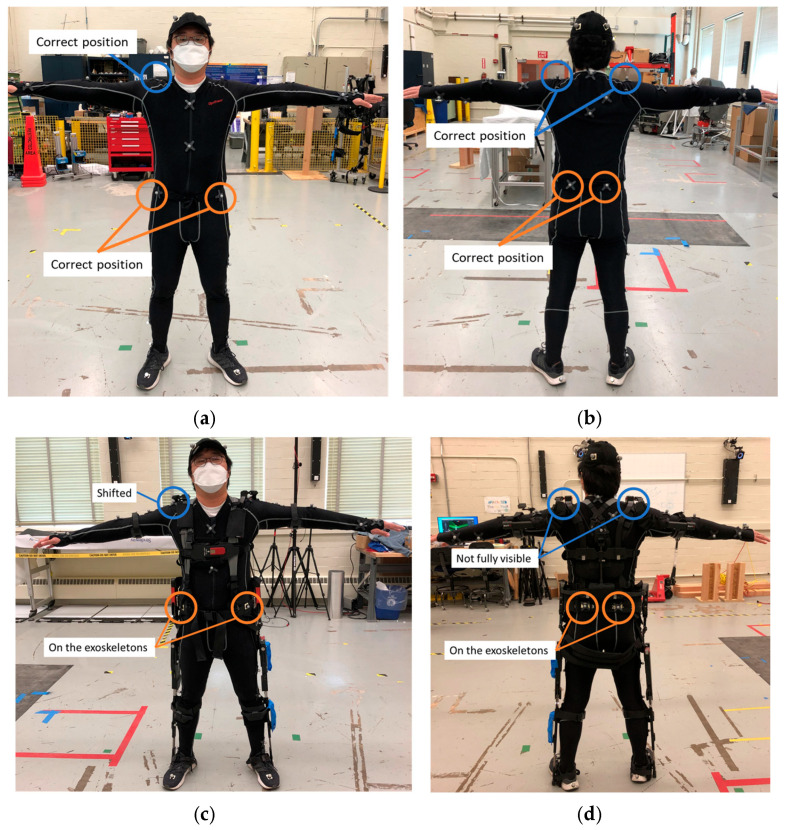
Example of the correct marker placements for the front (**a**) and back (**b**) of the control test and estimated marker placements for the front (**c**) and back (**d**) of the Type 1 exoskeleton test.

**Figure 18:**
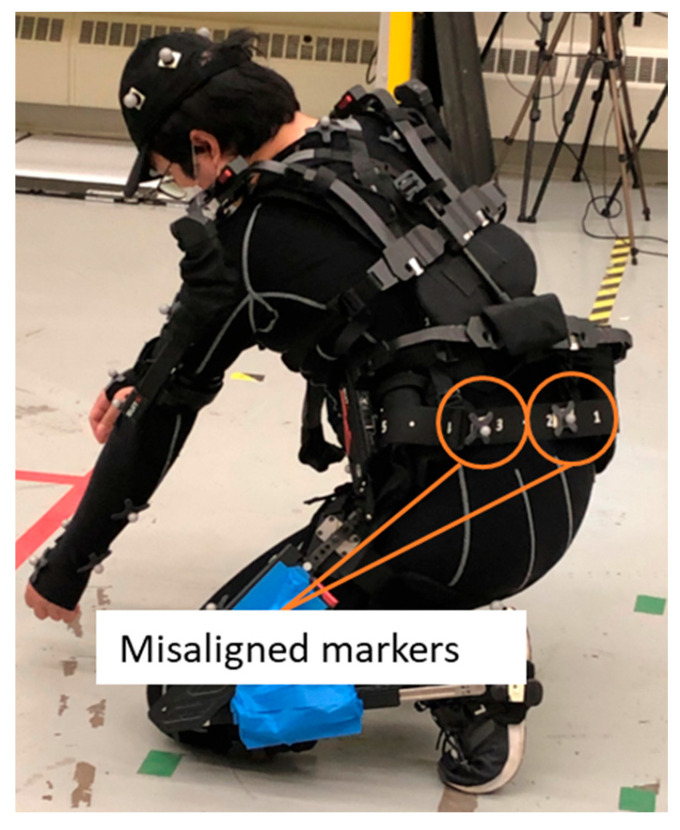
The markers on the exoskeleton misaligned with the human body.

An OTS can also be susceptible to occlusions from exoskeletons and industrial poses. Ideally, the optical markers are placed accurately and consistently at each anatomical landmark to build a subject’s skeletal tracking model and to initiate a pose estimation. Each exoskeleton type in this study had overlapping positions with the anatomical landmarks. To address the issue, the optical markers were placed on the exoskeleton, or the optical marker placement on the subject was shifted from the anatomical landmark. When the positions of the anatomical landmarks are translated from the ideal position, the OTS software either builds a model with inherent joint position and rotation errors or it fails to build the body model. When the marker is placed underneath the exoskeleton, the OTS fails to find the marker. In contrast, when the markers are placed on the exoskeleton, additional errors occur in tracking the human movement. Figure 17 shows the shifted marker placements to take into consideration the relationship of the exoskeleton to the body. Marker placement errors can occur from differences in body shapes and or misalignments between the human and the exoskeleton, as shown in Figure 18.

**Figure 19:**
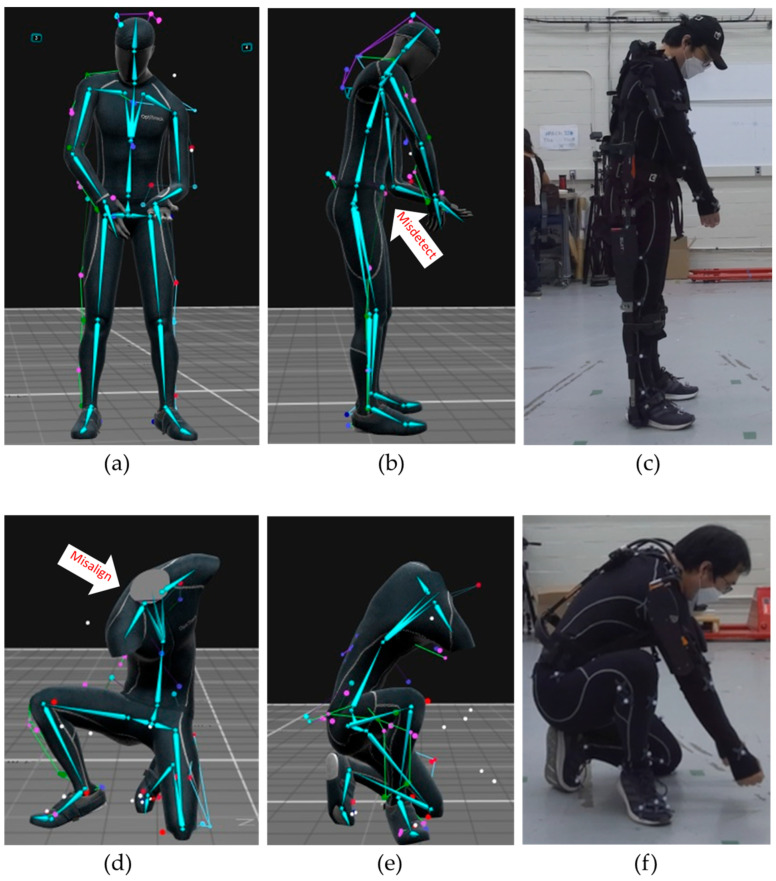
Unacceptable results from the Type 1 exoskeleton for pose 5 front (**a**) and side (**b**), and the actual pose from the side (**c**), and from the Type 2 exoskeleton for pose 14 (**d**–**f**).

The Type1 exoskeleton showed the lowest number of acceptable results, as it had the largest number of markers placed on the exoskeleton rather on the subject’s body. Therefore, most of the unacceptable results were caused by marker misalignments (U2). The Type 2 exoskeleton showed six unacceptable results due to having the second largest number of markers attached to the exoskeleton. Both the Type 1 and Type 2 exoskeletons showed multiple incorrect joint position and rotation estimations when the subject was in pose 14, possibly caused by the combined occlusions from the body and the exoskeleton. Figure 19 shows examples of unacceptable tracked skeletal models from the Type 1 and Type 2 exoskeletons. The Type 3 exoskeleton did not change the marker positions as much as the Type 1 and Type 2 exoskeletons. The reflective tapes on the Type 3 exoskeleton did not appear to affect the OTS pose performance. Similarly, rigid body pose measurements were acquired without a noticeable increase in uncertainty with prior use of retro-reflective tapes with at least 50 mm distance from the optical markers [50].

Table 12 shows ratios of acceptable to unacceptable results for monocular RGB camera-, depth camera-, and OTS-based pose estimation systems. All systems were affected by the subject pose. The OTS was affected only by the crouching arm-down pose, while the other systems were affected by additional poses. Proper placement of markers is key in using an OTS when wearing exoskeletons, but there are limitations in choosing the correct marker placement. For the OTS, missing joints were not observed, but incorrect body posture was observed several times. Monocular RGB camera- and depth camera-based systems showed decreased performance when wearing exoskeletons. Sources of human joint estimation errors from augmenting users with wearable robots include, but are not limited to, the exoskeleton frame, which can occlude the human joints; the exoskeleton joint, which can be erroneously detected as a human joint; and reflections, which can cause incorrect image capture. When joint estimation errors occurred from the monocular RGB camera- and depth camera-based systems, joints were missed or incorrectly detected, including cases where the whole body was not detected, as shown in Table 12 for the control and all exoskeletons. Comparisons between the monocular RGB camera-based and OTS-based pose estimation methods for other factors, including the static joint angle measurement, image capture performance, ease of implementation, or price, are discussed in the previous study [25].

### 4.3. Strategies for Exoskeleton Studies

This section discusses the strategies to use monocular markerless pose estimation systems in exoskeleton performance measurement studies. Experimental factors include the type of exoskeleton and the different task poses. Another factor is the selection of the camera position and distance relative to the subject. This paper provides the expected results according to the viewpoints for the given exoskeleton and the task poses. For example, top-down viewpoints are expected to show better results than straight viewpoints for RGB camera-based systems for side, side-back, and back views.

Defining joints of interest is also important. The joints of interest do not have to be the entire body in exoskeleton studies. By narrowing down the joints of interest, it is possible to collect more acceptable results. For example, U3 and part of U1 can be acceptable when the joints of interest are correctly estimated. If the missed or incorrectly estimated joints are extraneous, 140 can be added to A1 from U3, which is a 20% increase in the number of acceptable results for the Type 1 exoskeleton using the monocular RGB camera-based system (see Table 6).

Synchronized multi-view images can be useful for collecting the joint data separately. Although methods for improving joint estimation error by multi-view have not yet been found, as the current state-of-the-art methods for 2D and 3D pose estimation are based on joint-annotated training data [51], synchronized images can increase joint pose acceptability for further analysis. Each sensor can be assigned different joints of interest and capture partial parts of the body. The results can be combined to complete the joints of interest or used separately for the analysis. For example, for the Type 1 exoskeleton, the monocular RGB camera-based acceptable pose estimation results from a side straight viewpoint (in Table 9) could be increased to 91.4% when the joints of interest are the right arm and right leg, and where the left arm and left leg can be collected from a synchronized camera on the left side. The previous study successfully measured task completion time in peg-in-hole using the classification rule with defined joints of interest and synchronized multi-view strategies [12].

Classification rules to determine the joint pose acceptability depend on the purpose of the exoskeleton study. Typical study purposes include understanding how wearing an exoskeleton changes the task completion time, task preparation time, duration between tasks, task-performing pose, or task duration. Misalignments can be accepted when the task is defined by the relative poses between the joints. The classification rules become more stringent when the study requires clinically accurate joint poses.

As current pose estimation training datasets often do not include images of people wearing exoskeletons, algorithms based on these datasets may not be accurate for people performing tasks while wearing exoskeletons [25]. In addition, the newer pose estimation algorithms showed similar performance compared to the systems used in this study, as subjects with wearable devices may not be present in the models’ training sets [52]. Therefore, new models [53] may be created by including annotated images of people wearing exoskeletons [12] in existing training datasets. These annotations could be performed by creating a new neural network model in a software package such as DeepLapCut [54]. In addition, new pose estimation algorithms could be formulated to track individual points on both the human and the exoskeleton. These algorithms could be used to analyze the fit of an exoskeleton as well as track how well the exoskeleton aligns with the body while performing industrial tasks [53]. The algorithms could reduce incorrect joint estimation from occlusions by exoskeletons. Another area to extend the research and development of pose estimation algorithms is for clinical evaluations of exoskeleton use, such as gait pattern and gross motor function analysis [55].

## 5. Conclusions

This paper describes the observed effects of wearable robots on monocular markerless and multi-camera, marker-based joint position estimation systems for assessing an exoskeleton’s or exosuit’s impact while performing industrial tasks. Three types of exoskeletons were tested for 14 industrial task poses, 10 camera perspectives, and 3 pose estimation systems, including an OTS as the reference system. A total of 5577 trials were evaluated, where each trial included 100 frames of joint estimation results. Twenty-three trials were excluded due to technical errors, which resulted in missing data during data collection and processing. The acceptance rate of the result was 56% for the RGB, 22% for the depth, and 78% for the OTS body estimation systems.

The joint estimation performance decreased when the joints were within close proximity of each other, and in particular for poses that involved crouching. The performance was affected by the camera perspectives, mainly when a monocular camera was placed at an angle to the side, the side-back, and the back of the subject. The vertical monocular camera angles showed different effects depending on the task poses. Occlusions and other visibility constraints caused by the poses or the camera perspectives were considered the main factors causing joint detection errors. Wearing an exoskeleton contributed to additional errors in joint estimations due to the changes in the subject’s appearance, and additional occlusions caused by the exoskeleton frame. It was observed that the frame of the exoskeleton may be incorrectly detected as a body part, mainly the shoulder, the elbow, or the knee joints. The depth camera-based pose estimation system had issues resolving the reflective tape on the exosuit. As a comparison to the reference OTS, joint estimations based on the RGB camera system can provide useful information when assessing the relative impact that wearable robots may have on the user, but these systems may not have the accuracy or precision of a carefully designed OTS.

The limitations of this study come from uncertainties in collecting and classifying the precision of the human joint estimation results. During the three months of eight experiments, the RGB camera and depth camera were removed and relocated each day of the experiments. The distance and angle between the cameras and the subject were set with the best effort, but errors remain that may have affected the joint estimation results. There was also human error when classifying the results. The classification was performed by a single researcher to reduce inter-rater variability [56,57].

The main contribution of this study is a systematic methodology for evaluating and curating monocular markerless pose estimations in industrial exoskeleton studies. The results show how the quality of the joint identification changes depending on the subject’s pose and the camera’s position. Future studies could use the proposed method to determine camera positions, to design experiments to evaluate the impact of wearable robots on the user while performing industrial tasks, and to curate the images to be used for the analysis. The study also provided markerless pose estimation applications to assess the causes and effects of wearing an exoskeleton using three types of pose estimation systems. The potential evaluation metrics can be extended to wearable devices or other tools used for tracking human kinematics.

In future work, the study will be extended to more subjects as an additional variable via the exoskeleton performance dataset [44]. Pose estimation algorithms for subjects wearing exoskeletons can also be developed. This includes extending existing datasets to include people wearing exoskeletons as well as creating algorithms to track both human and exoskeleton joints when performing industrial tasks. The algorithms may then be applied to assess the performance of exoskeletons using markerless pose estimation systems for industrial tasks, including load positioning, load alignment, and assembly. In addition, pose estimation can also be applied to evaluate wearable robots for mobility tasks, including in confined spaces or on hurdles, beams, inclined planes, ladders, and stairs. The assessed performance will be compared to other measurement systems with statistical testing for verification. Furthermore, the pose estimation performance will be compared to state-of-the-art deep learning models. The study will be extended to biomechanical analysis such as human kinematics, balance, and posture. Further studies are also needed to evaluate the applications of markerless pose estimation algorithms for gait analysis.

## Figures and Tables

**Table 1:** Studies on pose estimation with wearable robots.

Sensor	Joint(s) of Interest	Subject Task	Measurement	Wearable Robot	Reference
OTS	Knee	Sit to stand	Human joint angle Exoskeleton joint angle	Non-powered full-body rigid	Bostelman 2019 [14]
RGB	Shoulder, wrist	Peg-in-hole	Joint poses	Non-powered full-body rigid	Virts 2022 [12]
OTS Force plate	Hip, knee, ankle	Gait	Joint angle Walking speed	Non-powered lower-body soft	Zhang 2022 [20]
Angle encoder	Hip, knee	Gait	Joint angle	Powered lower-body rigid	Yang 2020 [21]
Angle encoder IMU	Hip Trunk	Lift	Joint angle Kinematic	Powered lower-back rigid	Chen 2018 [22]
IMU	Hip, knee	Lift	Joint angle	Non-powered lower-back rigid	Pesenti 2022 [23]
IMU	Trunk, thigh, Shank, hip, knee	Squat	Joint angle	Powered knee soft	Yu 2019 [24]

**Table 2:** Studies on pose estimation performance measurement.

Sensor	Joint(s) of Interest	Pose or Task	Measurement	Factors Affecting the Performance	Reference
Depth OTS (reference)	Trunk, neck, shoulder, elbow, leg	Lowering a load Lifting a load Car assembly	Joint angle differences to reference system	Viewpoint (front, side-front) Occlusion	Plantard 2017 [30]
Depth	Head, pelvis, hand, foot	Reference pose: T-pose	Mean distance error of joints	Image resolution, body occlusions, subject–sensor distance	Romeo 2021 [31]
Depth	Full body	Reference pose: T-pose	Standard deviation of joint poses Counting undetected joints	Viewpoint (distance)	Tölgyessy 2021 [32]
Depth OTS (reference)	Full body	Gait	Joint pose differences to reference system	Subject walking speed	Albert 2020 [33]
Depth OTS (reference)	Ankle	Gait	Joint pose differences to reference system	N/A	Guess 2022 [34]
Depth OTS (reference)	Hip, knee, ankle	Gait	Joint angle differences to reference system	Viewpoint (0°, 22.5°, 45°, 67.5°, 90°)	Yeung 2021 [35]
Depth OTS (reference)	Shoulder	Shoulder flexion, abduction, internal rotation, external rotation	Interobserver reliability to reference system	N/A	Özsoy 2022 [36]
Depth OTS (reference)	Elbow, ankle	Arm swing Leg swing	Vertical coordinate trajectory	Occlusion	Yang 2017 [37]
RGB IMU (reference)	Hip and knee Ankle was excluded due to low performance	Gait	Joint angle differences to reference system	Viewpoint (back, side, side-back) Subject task pose—walking and running	D’Antonio 2021 [38]
RGB IMU (reference)	Hip, knee, ankle	Gait	Max/min joint angle differences to reference system	N/A	D’Antonio 2020 [39]
RGB OTS (reference)	Elbow, wrist, knee, ankle	Gait, jump, throw	Joint pose trajectory differences to reference system	Subject task pose	Nakano 2020 [40]
RGB Depth IMU (reference)	Full body	Six static poses for loading Four static poses for occlusion Dynamic simple lifting Dynamic complex lifting	Joint angle differences to reference system	Viewpoint (front, side, back) Occlusion	Kim 2021 [41]

**Table 3:** Subset of results for misdetection and misalignment for the three conditions tested.

	Condition 1 *	Condition 2	Condition 3
The joints are correctly detected.	100	0	12
One or two connected joints have misdetection.	0	37	88
One or two connected joints have misalignment.	0	63	0
Two or more independent joints have misdetection or misalignment.	0	0	0

* Condition 1: Type 2 exoskeleton, RGB straight back pose 3. Condition 2: Type 2 exoskeleton, RGB straight side-front pose 3. Condition 3: Control RGB, straight side-back pose 8.

**Table 4:** Acceptable and unacceptable pose estimation results.

Acceptability	Case	Description
Acceptable (A)		Pose estimation quality is sufficient for exoskeleton analysis when:
	1	Misdetections or misalignments are not observed
	2	One or two linked joints have misalignments
	3	One or two linked joints have misdetections for less than 20% of the samples
Unacceptable (U)		Pose estimation quality is insufficient for exoskeleton analysis when:
	1	Two or more unlinked joints have misdetections for more than 20% of the samples
	2	Two or more joints have misalignments for more than 20% of the samples
	3	One or two linked joints have misdetections for more than 20% of the samples

**Table 5:** Acceptability evaluation results of the front straight view for the control and Type 1 exoskeleton.

Exoskeleton	Image	Trial	Pose													
			1	2	3	4	5	6	7	8	9	10	11	12	13	14
Control	RGB	1	1	1	1	2	1	1	1	−1	2	1	−1	1	1	1
		2	1	1	1	1	1	2	1	−1	2	1	−1	1	1	3
		3	1	1	1	1	1	1	1	−1	−1	1	1	1	1	1
		4	1	1	1	1	1	1	1	−1	1	1	1	1	1	2
		5	1	1	1	1	1	1	1	−1	1	1	1	1	1	−3
	Depth	1	1	1	1	−1	3	1	1	−1	−3	1	−1	−3	1	−1
		2	1	1	1	−1	3	−3	1	−3	−3	1	−1	−3	−3	−1
		3	1	1	1	−1	3	1	1	−3	1	1	−1	3	3	−1
		4	1	1	1	−1	3	−3	1	−1	1	1	−1	−2	−3	−1
		5	1	1	1	−1	3	1	1	−1	3	1	−1	−1	3	−1
Type1	RGB	1	1	1	1	1	1	2	1	−1	1	1	1	3	2	−3
		2	1	1	1	1	1	1	3	−1	1	1	1	2	2	2
		3	1	1	1	1	1	1	1	−1	1	1	1	3	2	2
		4	1	1	1	1	1	1	1	1	1	1	1	3	2	2
		5	1	1	1	1	1	1	*	−1	1	1	1	3	2	2
	Depth	1	2	1	1	−1	2	1	−1	−1	−1	−1	1	−1	−1	−1
		2	2	1	1	−1	1	1	−1	−1	1	1	−3	−1	−1	−1
		3	2	1	1	−1	1	1	−1	−1	1	1	−3	−1	−1	−1
		4	−2	1	1	−1	1	1	−2	−1	1	1	3	−1	−1	−1
		5	−2	1	1	−1	1	1	*	−1	1	1	−3	−1	−1	−1

* An asterisk indicates missing data.

**Table 6:** Acceptability evaluation results summary by viewpoints.

Viewpoints		Total Evaluation		Acceptable Case (%)			Not Acceptable Case (%)				Missing Data (# of Occurrences)
			1	2	3	Total	−1	−2	−3	Total	
Straight	Front	558	55.6 *	10.9	3.0	69.5	20.3	4.3	5.9	30.5	2
	Side-front	558	61.6	9.9	4.3	75.8	10.0	0.2	14.0	24.2	2
	Side	558	15.1	4.8	4.1	24.0	28.1	3.2	44.6	76.0	2
	Side-back	550	6.0	11.6	0.7	18.4	42.2	0.2	39.3	81.6	10
	Back	560	13.9	7.3	0.0	21.3	66.3	7.3	5.2	78.8	0
Top down	Front	560	39.6	11.8	1.4	52.9	36.6	4.8	5.7	47.1	0
	Side-front	554	49.6	8.3	4.9	62.8	21.7	0.7	14.8	37.2	6
	Side	560	25.9	5.0	2.7	33.6	31.1	1.1	34.3	66.4	0
	Side-back	560	13.9	9.8	0.9	24.6	33.2	0.2	42.0	75.4	0
	Back	559	19.0	9.8	0.9	29.7	55.3	8.4	6.6	70.3	1

* Each cell represents the number of cases divided by the total number of evaluations.

**Table 7:** Acceptability evaluation results summary by image and exoskeleton type.

Image	Exoskeleton Type	Total Evaluation	Acceptable Case (%)				Not Acceptable Case (%)				Missing Data
			1	2	3	Total	−1	−2	−3	Total	
RGB	Control	700	44.1	14.0	3.9	62.0	15.3	1.3	21.4	38.0	0
	Type 1	696	39.1	14.1	3.7	56.9	20.0	3.0	20.1	43.1	4
	Type 2	700	43.6	12.6	0.7	56.9	14.3	2.6	26.3	43.1	0
	Type 3	693	39.7	15.3	1.4	56.4	13.6	4.3	25.7	43.6	7
Depth	Control	700	22.4	6.4	3.0	31.9	37.4	3.3	27.4	68.1	0
	Type 1	695	14.5	2.3	2.7	19.6	62.3	5.8	12.4	80.4	5
	Type 2	700	17.1	4.0	0.9	22.0	54.6	3.1	20.3	78.0	0
	Type 3	693	19.6	2.7	2.0	24.4	58.6	1.0	16.0	75.6	7

**Table 8:** Acceptable body estimation results for the exoskeleton type versus the pose.

Image	Exoskeleton	Pose (%)													
		1	2	3	4	5	6	7	8	9	10	11	12	13	14
RGB	Control	72.0 *	84.0	82.0	76.0	58.0	72.0	50.0	36.0	68.0	92.0	44.0	46.0	54.0	34.0
	Type 1	74.0	94.0	82.0	70.0	40.0	52.0	38.3	24.0	79.6	80.0	40.0	48.0	48.0	26.0
	Type 2	80.0	100.0	80.0	78.0	60.0	64.0	32.0	12.0	70.0	76.0	24.0	40.0	54.0	26.0
	Type 3	64.0	90.0	80.0	79.2	70.0	68.0	34.0	20.0	60.0	78.0	32.0	35.6	50.0	28.0
Depth	Control	54.0	58.0	62.0	40.0	46.0	42.0	36.0	10.0	28.0	50.0	0.0	2.0	18.0	0.0
	Type 1	22.0	42.0	52.0	30.0	40.0	26.0	2.1	0.0	14.3	36.0	8.0	0.0	0.0	0.0
	Type 2	32.0	50.0	54.0	30.0	42.0	20.0	4.0	2.0	34.0	24.0	0.0	8.0	8.0	0.0
	Type 3	40.0	54.0	44.0	39.6	42.0	34.0	20.0	0.0	26.0	38.0	2.0	0.0	0.0	0.0
	Total	54.8	71.5	67.0	55.3	49.8	47.3	27.2	13.0	47.5	59.3	18.8	22.3	29.0	14.4

* Each cell represents the number of acceptable results divided by the total number of evaluations (5 trials × 10 viewpoints = 50, and 400 in total, except missing data).

**Table 9:** Acceptable body estimation results by exoskeleton type and viewpoint.

Image	Exoskeleton	Straight Viewpoint (%)					Top-Down Viewpoint (%)				
		Front	Side-Front	Side	Side-Back	Back	Front	Side-Front	Side	Side-Back	Back
RGB	Control	87.1 *	100.0	38.6	30.0	30.0	67.1	91.4	62.9	55.7	57.1
	Type 1	92.8	95.7	37.7	**42.9**	14.3	**77.1**	81.2	50.0	35.7	42.9
	Type 2	84.3	97.1	27.1	34.3	38.6	71.4	74.3	57.1	35.7	48.6
	Type 3	72.9	100.0	28.6	33.8	35.7	57.1	91.2	54.3	34.3	55.7
Depth	Control	57.1	64.3	21.4	2.9	27.1	41.4	50.0	20.0	21.4	12.9
	Type 1	47.8	50.7	4.3	0.0	11.4	35.7	34.8	2.9	1.4	7.2
	Type 2	60.0	37.1	15.7	2.9	10.0	35.7	35.7	2.9	7.1	12.9
	Type 3	54.3	61.4	18.6	0.0	2.9	37.1	44.1	18.6	5.7	0.0
	Total	69.5	75.8	24.0	18.4	21.3	52.9	62.8	33.6	24.6	29.7

* Each cell represents the number of acceptable results divided by the total number of evaluations (5 trials × 14 poses = 70, and 560 in total, except missing data).

**Table 10:** Acceptable body estimation results for the viewpoint versus the pose.

Viewpoint		Pose (%)													
		1	2	3	4	5	6	7	8	9	10	11	12	13	14
Straight	Front	95.0 *	100.0	100.0	50.0	100.0	87.5	71.1	2.5	85.0	97.5	32.5	52.5	67.5	32.5
	Side-front	85.0	87.5	100.0	100.0	100.0	92.5	78.9	47.5	92.5	82.5	52.5	47.5	45.0	50.0
	Side	77.5	72.5	10.0	70.0	7.5	15.0	2.5	2.5	15.0	15.0	17.5	7.5	0.0	22.5
	Side-back	0.0	47.5	55.0	47.5	2.5	2.5	2.5	12.5	10.0	47.5	0.0	16.7	12.5	0.0
	Back	0.0	57.5	67.5	0.0	25.0	0.0	20.0	10.0	0.0	65.0	0.0	2.5	50.0	0.0
Top down	Front	42.5	90.0	100.0	100.0	100.0	50.0	0.0	0.0	77.5	82.5	10.0	45.0	35.0	7.5
	Side-front	100.0	100.0	85.0	100.0	90.0	87.5	37.5	15.0	60.5	50.0	37.5	40.0	50.0	30.0
	Side	52.5	30.0	2.5	52.5	30.0	72.5	57.9	17.5	57.5	62.5	37.5	0.0	0.0	0.0
	Side-back	62.5	80.0	62.5	37.5	0.0	37.5	7.5	5.0	25.0	27.5	0.0	0.0	0.0	0.0
	Back	32.5	50.0	87.5	0.0	42.5	27.5	0.0	17.9	52.5	62.5	0.0	12.5	30.0	0.0
	Total	54.8	71.5	67.0	55.3	49.8	47.3	27.2	13.0	47.5	59.3	18.8	22.6	29.0	14.3

* Each cell represents the number of acceptable results divided by the total number of evaluations (5 trials × 4 exoskeleton types × 2 image types = 40, and 400 in total, except missing data).

**Table 11:** Acceptable body estimation results of the OTS by exoskeleton type and pose.

Exoskeleton	Pose													
	1	2	3	4	5	6	7	8	9	10	11	12	13	14
**Control**	100.0 *	100.0	100.0	100.0	100.0	100.0	100.0	100.0	100.0	100.0	40.0	100.0	100.0	100.0
Type1	100.0	40.0	20.0	0.0	0.0	0.0	100.0	100.0	0.0	0.0	100.0	100.0	40.0	0.0
Type2	100.0	100.0	100.0	100.0	100.0	100.0	100.0	80.0	100.0	100.0	80.0	100.0	100.0	20.0
Type3	100.0	100.0	100.0	100.0	100.0	100.0	100.0	100.0	100.0	100.0	100.0	100.0	100.0	100.0

* Each cell represents the number of acceptable results divided by the total number of evaluations (5 trials per pose and exoskeleton).

**Table 12:** Ratio of acceptable to unacceptable results based on the exoskeleton type and the pose estimation system.

	RGB	Depth	OTS
Control	0.62	0.32	0.96
Type 1	0.57	0.19	0.43
Type 2	0.57	0.22	0.91
Type 3	0.56	0.24	1.00

## Data Availability

The original data presented in the study are openly available in NIST Data Repository at https://doi.org/10.18434/mds2-3143, Markerless Body Tracking System Results for Industrial Exoskeletons.
